# A comparison of rat models that best mimic immune-driven preeclampsia in humans

**DOI:** 10.3389/fendo.2023.1219205

**Published:** 2023-09-28

**Authors:** Fahmida Jahan, Goutham Vasam, Yusmaris Cariaco, Abolfazl Nik-Akhtar, Alex Green, Keir J. Menzies, Shannon A. Bainbridge

**Affiliations:** ^1^ Department of Biochemistry, Microbiology and Immunology, Faculty of Medicine, University of Ottawa, Ottawa, ON, Canada; ^2^ Interdisciplinary School of Health Sciences, Faculty of Health Sciences, University of Ottawa, Ottawa, ON, Canada; ^3^ Ottawa Institute of Systems Biology, University of Ottawa, Ottawa, ON, Canada; ^4^ Department of Cellular and Molecular Medicine, Faculty of Medicine, University of Ottawa, Ottawa, ON, Canada

**Keywords:** preeclampsia, disease subclasses, hypertension, immune, proinflammatory, TNF-α, Poly I:C, pregnancy

## Abstract

Preeclampsia (PE), a hypertensive pregnancy disorder, can originate from varied etiology. Placenta malperfusion has long been considered the primary cause of PE. However, we and others have showed that this disorder can also result from heightened inflammation at the maternal-fetal interface. To advance our understanding of this understudied PE subtype, it is important to establish validated rodent models to study the pathophysiology and test therapies. We evaluated three previously described approaches to induce inflammation-mediated PE-like features in pregnant rats: 1) Tumor necrosis factor-α (TNF-α) infusion via osmotic pump from gestational day (GD) 14-19 at 50ng/day/animal; 2) Polyinosinic:polycytidylic acid (Poly I:C) intraperitoneal (IP) injections from GD 10-18 (alternate days) at 10mg/kg/day/animal; and, 3) Lipopolysaccharide (LPS) IP injections from GD 13-18 at 20ug-70ug/kg/day per animal. Maternal blood pressure was measured by tail-cuff. Upon sacrifice, fetal and placenta weights were recorded. Placenta histomorphology was assessed using H&E sections. Placenta inflammation was determined by quantifying TNF-α levels and inflammatory gene expression. Placenta metabolic and mitochondrial health were determined by measuring mitochondrial respiration rates and placenta NAD^+^/NADH content. Of the three rodent models tested, we found that Poly I:C and LPS decreased both fetal weight and survival; and correlated with a reduction in region specific placenta growth. As the least effective model characterized, TNF-α treatment resulted in a subtle decrease in fetal/placenta weight and placenta mitochondrial respiration. Only the LPS model was able to induce maternal hypertension and exhibited pronounced placenta metabolic and mitochondrial dysfunction, common features of PE. Thus, the rat LPS model was most effective for recapitulating features observed in cases of human inflammatory PE. Future mechanistic and/or therapeutic intervention studies focuses on this distinct PE patient population may benefit from the employment of this rodent model of PE.

## 1 Introduction

Preeclampsia (PE) is a pregnancy associated disorder characterized by a sudden onset of hypertension at ≥20 weeks of gestation and evidence of maternal end organ dysfunction. It is a highly heterogenous condition in humans, with variable pathogenesis, timing of onset, maternal symptoms, and fetal outcomes – indicative of distinct subclasses of disease (1–8). Detailed clinical and placenta profiling by our group, and others, has identified the presence of a unique subclass of PE patients which demonstrate profound pro-inflammatory signalling at the maternal-fetal interface (1, 5, 6, 8, 9). Unlike the traditional dogma of PE pathogenesis, these patients do not demonstrate evidence of poor utero-placenta perfusion and hypoxia, or placenta up-regulation of anti-angiogenic factors (i.e. sFLT, sENG). Rather, placentas from these PE patients highly express pro-inflammatory cytokines and their receptors, such as tumor necrosis factor-α (TNF-α), interferon gamma (IFN-γ), TNF-α induced Proteins 2, 3, 6 (TNFAIP2, TNFAIP3, TNFAIP6), and interleukin 6 cytokine family signal transducer (IL-6ST). Further, these placentas demonstrate histopathology lesions consistent with chronic inflammatory insults, such as villitis of unknown etiology and/or massive perivillous fibrin deposition (1, 6, 7, 10). Clinically, this PE subclass demonstrates late pre-term deliveries (34-37 weeks) with milder maternal symptoms, however fetal growth is most significantly impacted in the subclass (1, 7).

Placenta mitochondrial dysfunction is a recognized contributor to placenta dysfunction across all PE subclasses and is thus an intriguing therapeutic target for this disorder (11–13). Interestingly, nicotinamide adenine dinucleotide (NAD^+^) consuming enzymes are highly and uniquely expressed in placentas from pregnancies with inflammatory PE – with NAD^+^ being a critical co-enzyme required for cellular reduction-oxidation (redox) status and homeostatic maintenance of energy metabolism and mitochondrial function (1, 14–17). These placentas likewise demonstrate a profound depletion of cellular NAD^+^ stores, suggesting a disruption of NAD^+^ signalling as a key mechanism through which mitochondrial function becomes disrupted in this subclass of PE (14). These results are not entirely surprising, given that several non-pregnant pro-inflammatory diseases (i.e non-alcoholic fatty liver disease, colitis, optic neuritis, central nervous system autoimmunity etc.) and ageing tissues demonstrate NAD^+^ depletion with paralleled mitochondrial impairment and organ dysfunction (15, 16, 18–23). Collectively, this set of data are important biological observations which hint at the molecular mechanisms through which placenta dysfunction may be established and/or propagated in this inflammatory PE subclass. However, further work focused on causal mechanisms of placenta disease in this understudied PE subclass is required, and as such animal model systems which can closely mimic the pathophysiology of this unique patient population are needed. Once established and validated, these models can help push forward the discovery of subclass (/etiology)-specific screening and treatment modalities for PE (1).

Spontaneous onset of PE is unique to humans and some non-human primates, however the establishment of animal models which mimic various aspects of PE pathophysiology have been established in dogs, sheep, rhesus monkeys and most frequently in rodents – mice and rats. Several comprehensive reviews (24, 25) on this topic nicely describe the most widely used of these models, including those established via induction of placenta ischemia, angiogenic imbalance (by increasing levels of anti-angiogenic factors such as s-FLT1, sENG) and genetic manipulations of the renin-angiotensin system (24, 25). In large part, these animal models lean heavily on a conceptual framework of PE pathogenesis that included poor utero-placenta perfusion and placenta hypoxia as central features. These well-established models certainly demonstrate utility in expanding our understanding of human PE with these same features (i.e. the canonical PE subclass), however their utility is likely limited for explorations of the smaller and less characterized subclasses of PE pathophysiology, including the inflammatory PE subclass described.

There are, however, a handful of immune-mediated, non-genetic animal models of PE that have been established, which may effectively serve this purpose. These models have been established in rodents, generated via continuous TNF-α infusion or daily injections of Toll-like receptor (TLR) agonists across pregnancy (e.g. lipopolysaccharide- LPS, Polyinosinic:polycytidylic acid- Poly I:C). TNF-α is an important mediator of inflammation in human PE (1, 26, 27), with well described elevations in serum and placenta (1, 27, 28). TNF-α infusion in pregnant rats from gestational day (GD) 14 to 19 has been reported to successfully induce maternal hypertension, with no described changes to litter size or fetal weight (29). Alternatively, mouse and rat models of PE have been established via TLR agonist activation, as TLR signalling cascades have been implicated in placenta inflammation described in human cases of PE (30). In this vein, some groups have carried out daily injections of LPS to illicit an immune response. LPS, a bacterial cell-wall component, is a potent inducer of TLR-4 mediated inflammatory cascade (31) and is proposed to induce PE-like symptoms in this model through TNF-α mediated trophoblast dysfunction and maternal systemic inflammation (24, 32–34). As with TNF-α infusion, LPS administration to rats during pregnancy increased maternal blood pressure, however decreased fetal growth and survival was also described in this model (32, 35–38). Several other TLR agonists have been tested, including Poly I:C – a TLR-3 agonist; Imiquimod (R837) - a TLR-7 agonist; CLO97 (Imidazoquinoline compound) – a TLR7/8 agonist; and synthetic CpG oligonucleotide (ODN 2395)- a TLR-9 agonist. In all cases, treatment with these agonists resulted in increased maternal blood pressure with variable reported effects on fetal survival and growth (30, 39, 40).

In the current study, three previously published inflammation-mediated rodent models of PE were evaluated for their potential utility in future investigations of the inflammatory subclass of PE. Specifically, rat PE models established in the following ways were directly compared: 1) TNF-α infusion via osmotic pump, 2) Poly I:C treatment by intraperitoneal (IP) injection, and 3) LPS treatment by IP injection. PE-like features (i.e. maternal hypertension), fetal health outcomes, evidence of chronic placenta inflammation and metabolic dysfunction were compared across all models. This study will serve as a solid resource for other groups aiming to establish an appropriate *in vivo* animal model of PE to investigate underlying mechanisms of disease, biomarker discovery or therapeutic interventions for the inflammatory subclass of PE.

## 2 Materials and methods

### 2.1 Model establishment and pregnancy outcomes

All animal studies were performed in accordance with University of Ottawa animal care ethics and guidelines (protocol# HS2923). Sprague Dawley rats were purchased from Charles River Laboratories International, Inc. Animals were housed in a room with stable temperature of 23°C and a 12:12-hr light-dark cycle was maintained. Vaginal smears were performed to confirm estrus for timed-mating (41). Body weights were recorded each day to monitor pregnancy progression. Animals were allocated to one of the model treatment groups described below.

- TNF-α model (n=10): Recombinant rat TNF-α protein (510-RT-010, R&D systems) was infused at 50ng/day/animal concentration by implanting osmotic mini pump (Alzet Model 2001) on GD 13 (infusion days GD 14-19), as previously described (29, 42).
- Poly I:C model (n=10): Polyinosinic–polycytidylic acid sodium salt (P1530-100MG, Sigma-Aldrich) was solubilized in saline and injected intraperitoneally (IP) every alternate day between GD 10-18 at 10mg/kg/day, as previously described (39).
- LPS model (n=10): Lipopolysaccharides from Escherichia coli O55:B5 (L2880-10MG, Sigma-Aldrich) was solubilized in saline and injected by IP from GD 13-18 at 20ug-70ug/kg/day incremental dose, as previously described (35).
- Saline control (n=10): Controls were randomly allocated for saline osmotic pump infusion or saline IP injections. (n=3 osmotic pump, n=3 IP from GD10-18 alternate days and n=4 IP from GD 13-18)

Necropsy of pregnant rats was performed on GD 19, with collection of litter size, number of resorption sites and fetal and placenta weights.

### 2.2 Maternal blood pressure measurement

For all models, blood pressure (BP) measurements were performed using tail-cuff plethysmography using the CODA® high-throughput non-invasive system (Kent Scientific Corporation). Measurements were recorded from GD 15-19. Animals were acclimatized to the tail-cuff system for 4 days prior. On the day of recording, 5-10min was allowed for body temperature stabilization and a continuous 20min blood pressure recording was taken. The collected BP measurements for the TNF-α model using this method did not align with previous reports, and as such BP measurements were confirmed using the radiotelemetry method (HD-S10 Implant, Data Sciences International, USA) in a subsequent set of TNF-α treated rats (n=4; 50ng/day, GD14-19) and saline treated controls (n=4). In this subset, 10 days prior to mating, animals were subjected to surgery and a blood pressure transducing catheter was place into the femoral artery and the transmitter body was placed subcutaneously. A detailed method of the surgery is described by Huo et. al, 2014 (43).

### 2.3 Placenta histomorphometry

Rat placentas were bisected at the point of umbilical cord insertion, fixed in 4% PFA [in phosphate buffer saline (PBS)], paraffin-embedded, sectioned at 4um thickness and stained with hematoxylin and eosin (H&E). The slides were scanned using a Zeiss Axio Scan Z1 slide scanner, and the images were analyzed using the ZEISS ZEN lite software. The placenta diameter was determined by drawing a straight line perpendicular to the umbilical cord insertion and extending to both placenta extremities. Similarly, to estimate the depth of the decidua, junctional zone, and labyrinth placenta layers, three lines located in the middle, right lateral, and left lateral regions of the placenta were drawn perpendicular to the placenta diameter line in each layer. The results were averaged and expressed in micrometers (um).

### 2.4 Albumin analysis in the urine

Urine albumin levels was measured as a proxy for kidney function assessment using Rat Albumin ELISA kit according to manufacturer’s instruction (Abcam, ab108789).

### 2.5 Placenta inflammation

Cluster of Differentiation 68 (CD68) staining, a marker of macrophage, has been carried out on rat placental sections (44). Placental sections were prepared as mentioned above (2.3). Antigen retrieval was performed with citrate buffer (10mM, pH 6.0) in microwave for 7 mins. Sections were treated with 3% H_2_O_2_ in PBS for 30 minutes to block endogenous peroxidase. Sections were then blocked with 1% BSA in PBS for 1h at room temperature to prevent non-specific protein binding. Diluted CD68 Monoclonal Antibody made with 1% BSA in PBS (eBioscience™, 14-0681-82) was added to the slides and incubated at 4°C overnight. Slides were washed and incubated with biotinylated secondary antibody, (Invitrogen, A18919) for 1 hour at room temperature. Slides were then incubated with streptavidin-HRP for 30 mins (DAKO, K0675). Finally, 3,3′-Diaminobenzidine (DAB) plus H2O2 was used to develop the reaction (Sigma Aldrich, D5637). Slides were counterstained with Harris hematoxylin. Images were taken with a Zeiss Axio Scan Z.1 scanner and QuPath software was used for analysis using the positive cell detection tool (45).

Quantikine® Rat TNF-α Immunoassay immunosorbent assay (R&D Systems, Minneapolis, MN) was used for measuring placenta TNF-α protein levels. 40 mg of placenta tissue was homogenised in PBS-EDTA 5mM extraction buffer containing protease inhibitor cocktail (complete™ Roche 11836170001) and the immunoassay was performed according to manufacturer’s instruction.

RNA was extracted to assess gene transcript expression of key pro-inflammatory signalling mediators and growth factors previously linked to placenta inflammation (46–52) [e.g. *Tnf-α, Ifn-γ*, placental growth factor *(Pigf)*, chemokine ligand 2 *(Ccl2)*, inducible nitric oxide synthase *(inos or Nos2)*, intercellular ddhesion molecule 1 *(Icam-1)* and macrophage migration inhibitory factor *(Mif).* TRIzol (invitrogen) was used to extract total RNA from rat placenta, with RNA concentrations measured using NanoDrop technology (Thermo Fisher Scientific). Subsequently, gDNA was eliminated and 1µg of total RNA was transcribed to cDNA with iScript™ gDNA Clear cDNA Synthesis Kit (Bio-Rad; #1725035). In a 20µL reaction, SsoAdvanced Universal SYBR Green Supermix was used to amplify cDNA following the manufacture’s instructions (Biorad). Selected gene expression was analyzed in triplicate with SYBR green chemistry in a CFX384™ Real-Time PCR Detection System. Then, using comparative cycle threshold method (ΔΔCq), quantification cycle (Cq) values were normalized based on beta-actin expression. Primer sequences used for qPCR can be found in the **Supplementary File 1**.

Maternal systemic inflammation was determined by measuring plasma CCL2 levels using Rat CCL2/MCP1 ELISA kit according to manufacturer’s instruction (Proteintech, KE20009).

### 2.6 Placenta mitochondrial function

High-resolution oxygraphy was used to assess oxygen respiration rates of mitochondria isolated from fresh rat placenta samples. Isolation of mitochondria was performed according to Frezza et al. with slight modifications as noted below (53). Animals were decapitated (54) and 6-8 placentas/litter were quickly removed and pooled, washed in cold PBS and placed in ice cold buffer A [300mM sucrose mM, 10mM Tris-HCl, 1mM EGTA-Tris Base and 0.1% bovine serum albumin (BSA, fatty acid-free); pH 7,2]. Placentas were first cut into large pieces and washed twice with buffer A to remove blood. These samples were then minced in a small volume of buffer A. The sample was transferred to a 30ml Potter-Elvehjem homogeniser, topped up with buffer A, and homogenised at 500rpm for 6 strokes. The suspension was centrifuged at 1000Xg and 4 °C for 10 min. The supernatant was collected in a new tube and the centrifugation step was repeated. To omit floating fat layers, a syringe was used to collect the supernatant. The combined supernatant was transferred to a new tube and centrifuged at 8000Xg and 4 °C for 10 min. Following aspiration of the supernatant, buffer B (300mM sucrose, 10mM Tris-HCl and 0.05mM EGTA-Tris Base; pH 7), was added to the pellet and gently mixed by pipetting, followed by centrifugation at 8000Xg and 4 °C for 10 min. This step was repeated. The resulting mitochondrial pellet was re-suspended in 200μl of MiR05 respiration buffer (110 mM D-sucrose, 60 mM lactobionic acid, 20 mM taurine, 20 mM HEPES, 10 mM KH2PO4, 3 mM MgCl2, 0.5 mM EGTA, 1 g/L fatty-acid free BSA; pH 7.1 at 23°C). An aliquot was taken from the mitochondrial suspension to perform detergent compatible colorimetric (DC) protein assay (Biorad) to quantify protein concentration.

Oxygen consumption rate was recorded using Clark-type electrodes according to a previously published protocol (55). 2 mg/ml of mitochondrial protein content was added to the MiR05 respiration buffer. Baseline traces were recorded, and mitochondrial substrates and inhibitors were injected in sequence: i) 5mM glutamate and 2,5mM malate were added as a substrate for Complex-I mediated oxygen consumption, ii) ADP (1 mM) was added to record ADP stimulated oxygen consumption through Complex-I or Complex-I driven state-III respiration, iii) 1 mM amytal was added to inhibit Complex-I mediated oxygen consumption, iv) 5mM succinate was added to record Complex-II mediated oxygen consumption or Complex-II driven state-III respiration v) 5 μM antimycin A was added to inhibit Complex-III mediated electron transfer in order to measure complex-IV mediated oxygen consumption or Complex-IV driven state-III respiration vi) Tetramethyl-p-phenylenediamine (TMPD)/Ascorbate (5/0.3 mM), was added to record Complex-IV mediated oxygen consumption, and vii) Potassium cyanide was added to stop oxygen consumption by mitochondria.

### 2.7 Placenta metabolic signalling

Placenta NAD^+^ and NADH was quantified using a Biovision NAD^+^/NADH kit (K337). 30-40mg of pooled (by litter) pulverized placenta tissue was added to 300ul of extraction buffer. Tissues were further homogenized using 21-gauge syringe needles. Quantification was performed according to the manufacturer’s instructions.

### 2.8 Statistical analysis

All statistical analyses were performed using GraphPad Prism software (Version 9.5). All values are reported as mean ± standard deviation (SD). Comparison was performed between saline control and treatment groups using a one-way ANOVA with a Fisher Least Significant Difference (LSD) test and a p value of < 0.05 was considered statistically significant. For fetal survival/death analysis and BP measurement a 2-way ANOVA with Fisher LSD test was applied. For fetal and placental weight measurements, a mixed model effect analysis with Fisher LSD test was applied to consider fetal/placental weights in relation to litter.

## 3 Results

### 3.1 Inflammatory PE-like features and pregnancy outcomes were most consistently demonstrated in the LPS rat model

**Figure 1:**
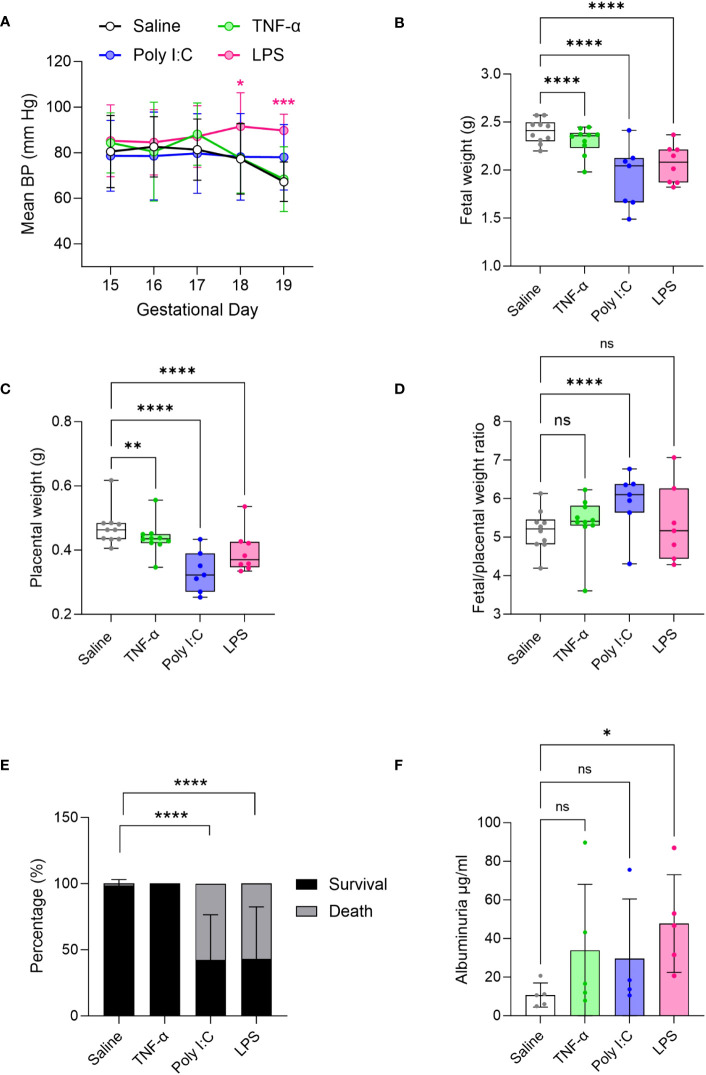
Evaluation of preeclampsia (PE)-like features in TNF-α, Poly I:C and LPS treated rat models of inflammatory PE. **(A)** Mean blood pressure by tail-cuff system measured from gestational day 15-19 in treated TNF-α (green), Poly I:C (blue) and LPS (pink) pregnant rats. n=8 pregnant rats/treatment group. **(B-D)** Fetal, placenta weight and its ratio. n=10 litters/treatment group; n=1-16 pups or placentas/litter. **(E)** Average fetal survival and death (percentage) in each treatment group at GD 19. **(F)** Urinary albumin levels in the pregnant rats at GD 19. n=4-5 pregnant rats/treatment group. *P<0.05,**P<0.01,***P<0.001, and ****P<0.0001. Error bar indicates standard deviation (SD). ns= non-significant, Tumor necrosis factor (TNF-α), Polyinosinic:polycytidylic acid (Poly I:C) and Lipopolysaccharide (LPS).

As development of hypertension during pregnancy is a key clinical feature of PE (56), we measured BP in the pregnant rats using a tail-cuff blood pressure system. Results show that mean BP was significantly increased only in LPS treated pregnant rats compared to saline treated controls (**Figure 1A**). In our hands, neither Poly I:C or TNF-α treatments showed an effect on BP. Given that TNF-α infusion was previously shown to increase BP by GD 19 (29, 42), we set out to confirm our TNF-α tail-cuff data using radio-telemetry, considered the gold standard of BP measurement techniques. Similar to tail-cuff results, radio-telemetry of TNF-α treated pregnant rats exhibited no change in BP compared to their corresponding controls (**Supplementary Figure 1**). Fetal and placenta growth was decreased in all treatment models, compared to saline controls (**Figures 1B, C**). Fetal/placental ratio did not change with TNF-α or LPS treatment, however, with Poly I:C treatment there was an increase in fetal/placental ratio (**Figure 1D**). Poly I:C and LPS treatment models also demonstrated decreased fetal survival, with increased number of resorption sites at GD19, whereas no fetal losses were observed in the TNF-α group (**Figure 1E**). An indirect assessment of kidney function via urine albumin measurements suggested that only LPS treatment negatively impacted kidney function (**Figure 1F**).

### 3.2 Placenta morphometry was altered in LPS, Poly I:C and TNF-α treated pregnant rats

**Figure 2:**
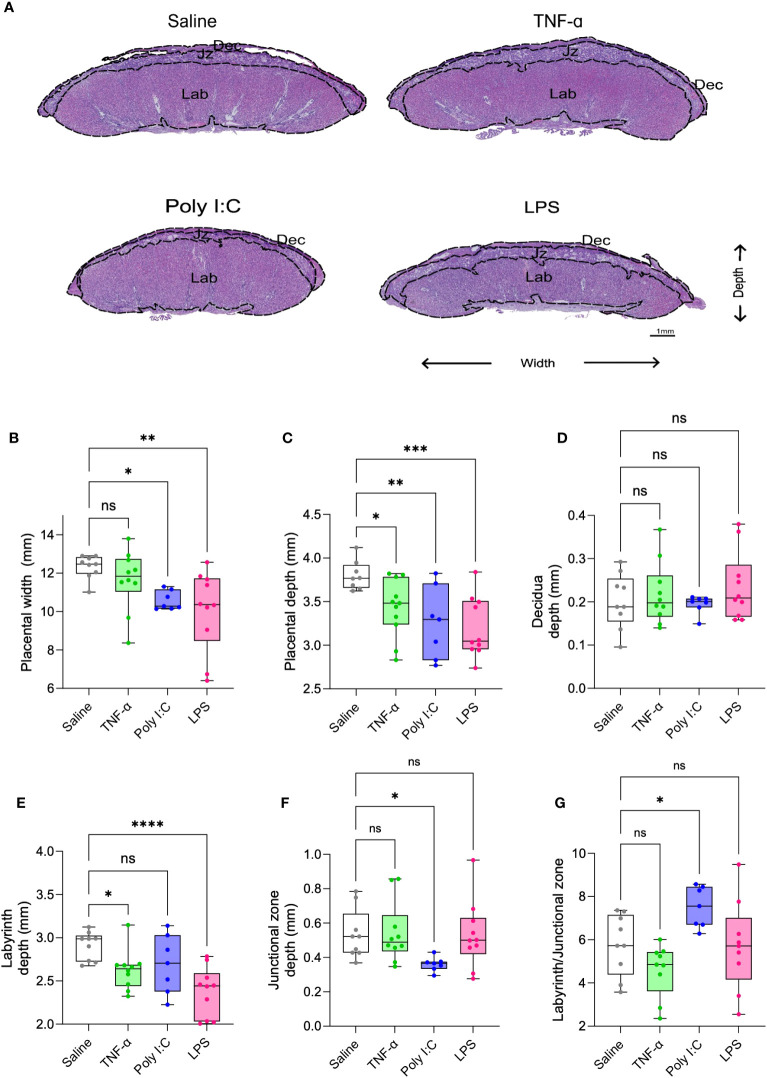
Placenta morphometry in TNF-α, Poly I:C and LPS treated pregnant rats. Representative images of placenta cross-section from saline control (white), TNF-α (green), Poly I:C (blue) and LPS (pink) treatment groups **(A)**. Measurement of placenta width **(B)**, depth **(C)**, decidua **(D)**, labyrinth **(E)**, junctional zone **(F)**, and labyrinth/junctional zone ratio **(G)** on H&E stained sections. n=9-10 litters/treatment group; n=1 placenta/litter. *P<0.05, **P<0.01, ***P<0.001, and ****P<0.0001. Error bar indicates standard deviation (SD). ns= non-significant, Tumor necrosis factor (TNF-α), Polyinosinic:polycytidylic acid (Poly I:C) and Lipopolysaccharide (LPS).

H&E-stained placenta cross-sections were used for histomorphology assessments. Placentas from Poly I:C and LPS treatment groups demonstrated a reduced placenta width and depth (**Figures 2A-C**). TNF-α treatment led to reduction only in the placenta depth. When determining the effect on individual layers of placenta, none of the treatments had any effect on the decidual depth (**Figure 2D**). Placentas from both TNF-α and LPS treatment group demonstrated a reduction in the depth of the labyrinth (exchange region of the placenta), with LPS treatment having more pronounced impact (**Figure 2E**). Whereas placentas from Poly I:C treated rats showed junctional zone depth deficits (**Figure 2F**). Labyrinth to junctional zone ratio was significantly increased in the Poly I:C group while the ratio did not change with TNF-α or LPS treatment (**Figure 2G**). Representative higher resolution H&E images of the placenta suggests that TNF-α treated rats did not show major histological changes in the placentas when compared to control rat placentas. While Poly I:C treatment led to mild histological changes in the placenta, mainly evidenced by trophoblast barrier thickening in the labyrinth, LPS treatment caused visible degeneration of the trophoblast and pronounced accumulation of immune cells in the labyrinth (**Supplementary Figure 2**).

### 3.3 LPS treatment induces immune cell recruitment, TNF-α protein expression and inflammatory gene expression in the placenta

**Figure 3:**
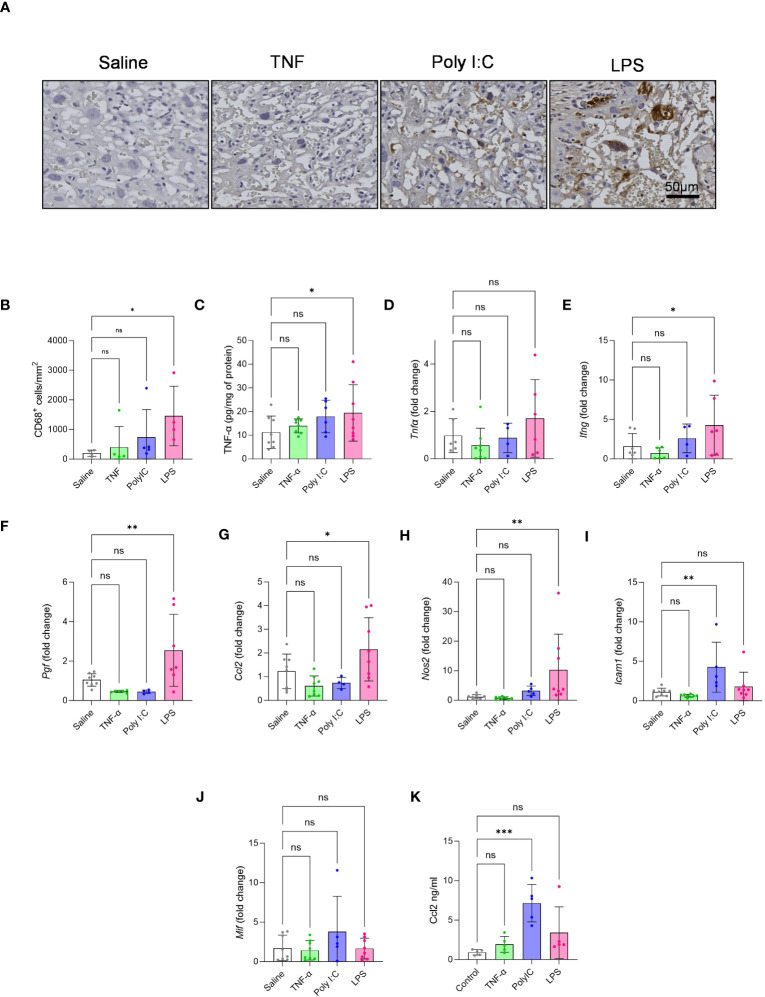
Effect of TNF-α, Poly I:C and LPS treatment in mediating placenta inflammation. **(A, B)** CD68 macrophage staining was performed on placental sections to quantify the number of infiltrated macrophages in the labyrinth of the placenta. n=4-5 litters/treatment group, n=1 placenta/litter. **(C)** Measurement of placenta TNF-α protein by ELISA. **(D-J)** Gene expression of *Tnf-α, Ifn-γ, Pigf, Ccl2, Nos2* (iNOS), *Icam-1* and *Mif* by qPCR. n=4-8 litters/treatment group, n=1 placenta/litter. **(K)** Maternal plasma CCL2 levels by ELISA. n=5 pregnant rats/treatment group *P<0.05, **P<0.01, and ***P<0.001. Error bar indicates standard deviation (SD). ns= non-significant, Tumor necrosis factor (TNF-α), Polyinosinic:polycytidylic acid (Poly I:C) and Lipopolysaccharide (LPS).

CD68 staining for the detection macrophage infiltration in the placenta labyrinth showed that only LPS treatment led to a significant increase in the number of CD68^+^ cells in the labyrinth (**Figures 3A, B**). Gene expression patterns associated with TNF-α mediated pro-inflammatory signalling have been described in placentas from patients with immune-driven PE (1, 7). Thus, we measured placenta TNF-α protein levels by performing an ELISA. We found that only LPS treated rats had significantly higher placenta TNF-α protein levels when compared to our saline control (**Figure 3C**). We further measured placenta gene expression for a set of pro-inflammatory and growth factor mediators (**Figures 3D–J**). Only the placentas from the LPS treatment model demonstrated a pattern of gene expression consistent with pro-inflammatory signalling, including elevated expression of *Ifn-γ*, *Pigf*, *Ccl2* and *inos* (**Figures 3E-H**). Whereas Poly I:C treated placentas had significantly higher gene expression of *Icam-1* (**Figure 3I**). *Mif* expression, on the other hand, did not alter in any treatment groups (**Figure 3J**). We also measured maternal systemic inflammation by measuring plasma CCL2 levels. Results indicate that Poly I:C induced robust production of CCL2 systemically while LPS showed a similar trend (**Figure 3K**).

### 3.4 Placenta mitochondrial function and NAD(H) redox status were most adversely impacted by LPS treatment in pregnant rats

**Figure 4:**
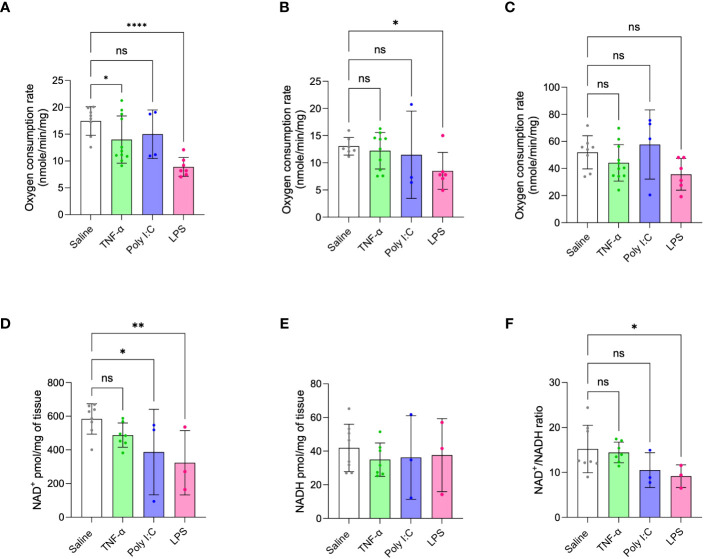
Placenta mitochondrial function in TNF-α, Poly I:C and LPS treated pregnant rats. **(A-C)** Mitochondrial oxygen consumption rates were measured by Oxygraph system. Mitochondrial complex-I, II and IV driven state-III respiration rates were determined. n=4-9 litters/treatment group, n=6-8 pooled placentas/litter. **(D-F)** Placenta NAD^+^/NADH levels were measured and their ratio was determined. n=3-8 litters/treatment group, n=1 placenta/litter. *P<0.05, **P<0.01 and ****P<0.0001. Error bar indicates standard deviation (SD). ns= non-significant, Tumor necrosis factor (TNF-α), Polyinosinic:polycytidylic acid (Poly I:C) and Lipopolysaccharide (LPS).

Oxygen respiration rates of isolated placenta mitochondria were measured and compared across all treatment models, given that metabolic dysfunction is a signature of placenta dysfunction in human PE (11–13). Consistent with the ability of LPS treatment to replicate multiple phenotypes of inflammation-mediated PE, this model also demonstrated decreased placenta state-III respiration or oxygen consumption rates through mitochondrial complex-I and complex-II. TNF-α treatment showed a moderate decrease in complex-I driven state-III respiration or oxygen consumption rates and Poly I:C treatment did not affect placenta mitochondrial respiration at all (**Figures 4A, B**). Mitochondrial complex-IV activity did not change for any of the treatment groups (**Figure 4C**). Altered levels of NAD(H) redox status is a strong marker of mitochondrial dysfunction and impaired cellular redox status (14, 15, 18). Moreover, decreased placenta NAD^+^ level is a unique feature of human inflammatory PE subclass (14). Thus, we determined the ratio and content of placenta NAD^+^ and NADH. Placentas from LPS treated rats showed decreased NAD^+^ content and NAD^+^/NADH ratio compared to saline control placentas (**Figures 4D-F**). Poly I:C treatment only exhibited a decrease in placenta NAD^+^ levels, while TNF-α treatment had no effect.

## 4 Discussion

The current study was undertaken to evaluate the utility of three previously described immune-mediated rodent models of PE (29, 35, 39, 42), determining which of these models may be best suited for future investigations of the inflammatory PE subclass described recently in human populations (1, 7). Collectively, the PE-like model established by daily IP injections of LPS from GD 13-18 most closely mimicked clinical and pathological findings described in human cases of inflammatory PE, including the establishment of maternal hypertension, evidence of proteinuria, fetal growth restriction and morphometric and functional evidence of inflammation-mediated placenta dysfunction. Conversely, the other two models examined (Poly I:C injection and TNF-α infusion) demonstrated some, but not all, key features attributed to this unique PE subclass and as such may not be as well-suited for future investigations of inflammatory-driven PE.

Maternal hypertension is the keystone finding of PE and is a necessary feature for any PE-like rodent model. In our study, only LPS treated pregnant rats showed elevations in maternal BP across pregnancy (**Figure 1A**). Our data suggests that LPS treatment led to macrophage infiltration in the placenta and induced expression of several pro-inflammatory mediators, such as TNF-α, IFN-γ, PGF, CCL2 and iNOS – all critical for sustaining inflammatory signalling (**Figures 3A-H**) (48, 50–52, 57–59). Moreover, it showed a non-significant trend of increases plasma CCL2 levels (p value 0.0792), likely indicating some level of systemic inflammation. We think that together, such a robust inflammatory stimulus may significantly contribute to maternal endothelial dysfunction, resulting in hypertension (32, 52, 57–60). This finding is consistent with other PE studies utilizing LPS model (32, 35–38). On the other hand, Poly I:C treatment showed a non-significant trend towards increased BP on GD 19. This is aligned with our finding that Poly I:C treatment could only increase the transcript levels of ICAM-1 but not the other pro-inflammatory genes tested (**Figure 3**). Poly I:C, however, showed higher levels of maternal plasma CCL2 protein levels, but interestingly did not demonstrate increased placenta *ccl2* mRNA levels (**Figure 3**). This is consistent with another study in which 20 mg/kg subcutaneous injection of Poly I:C in pregnant mice led to maternal plasma CCL2 increase without increasing placental *Ccl2* mRNA (61). It may be that Poly I:C impacts CCL2 levels post translationally or Poly I:C treatment may be inducing a larger production of CCL2 in other maternal organs (not the placenta), being then released into the bloodstream/plasma. Nonetheless, our data suggest that Poly I:C mediated maternal systemic inflammation does not induce similar inflammation at the level of placenta. In human inflammatory PE patients, we currently do not know the proinflammatory cytokine profile of the maternal plasma/blood. But placental gene expression and histopathology indicates aberrant placental inflammation which is prominently evident in the LPS model. On the other hand, the TNF-α treatment in the pregnant rats had no effect on maternal BP when measured via tail cuff (**Figure 1A**). Previous reports describe an induction of hypertension in this rat model – using the same dosage (50ng/day/animal) and exposure time of the current study (29, 42). To confirm the discrepancy in results, we performed radio-telemetry on an additional subset of TNF-α-treated pregnant rats, and controls. In line with our tail-cuff data, BP measurements via radio-telemetry likewise demonstrated no significant increase in BP with TNF-α infusion (**Supplementary Figure 1**). When looking at placental infiltration of macrophages and gene expression for markers of pro-inflammatory signalling in the TNF-α treated rats, surprisingly no evidence of placental inflammation was observed, indicating that this treatment was not sufficient to illicit inflammation-mediated placental dysfunction or systemic hypertension (**Figure 3**). It is possible that treatment with higher concentrations of TNF-α may be needed in order to see this type of pathology.

The placental dysfunction established in human cases of inflammatory PE profoundly impacts fetal and placenta growth trajectories, more so than observed in the other two described PE subclasses (1). All three models tested here likewise demonstrated evidence of fetal and placental growth restriction (**Figures 1B, C**). While Poly I:C and LPS treatments led to significant fetal loss, TNF-α treatment had no negative effect on fetal survival (**Figure 1E**). When examining the types of placental defects established in these models (**Figures 2A-G**), Poly I:C treatment appeared to have the largest impact on the junctional zone (JZ). JZ produces hormones, growth factors and cytokines, thus, regarded as a critical endocrine region of the rodent placenta. Further, this region of the placenta serves as an energy reserve through glycogen storage (62). In several different rodent models, JZ defects have been linked to fetal growth restriction via dysregulated hormone production – particularly those hormones required to support utero-placenta and feto-placenta angiogenesis (63, 64). In the current study no significant differences in size of the vascularized labyrinth region were observed in the Poly I:C model. Intriguingly, the higher magnification images of these placenta sections (**Supplementary Figure 2**) suggest barrier thickening of labyrinth which led to a higher proportion of labyrinth compared to JZ (**Figure 2G**). This is consistent with increased fetal/placental ratio in the Poly I:C group where placental transport efficiency is increased in an attempt to compensate for fetal growth (**Figure 1D**). In this study, however, in-depth morphometric analysis of utero-placenta and feto-placenta vascular structures and/or angiogenic signalling were not specifically evaluated. As such, we cannot rule out this mechanism in the development of fetal growth restriction in this model. It should be noted, however, that deficits in placenta angiogenic and/or vasculogenic pathways have not been reported to date in human cases of inflammatory PE, and therefore this rodent model may not best replicate the placenta deficits observed in human populations. In contrast, in the tested LPS model, the most prominent placenta defects observed were in the labyrinth exchange region, coupled to increased number of CD68^+^ macrophages and elevated expression of numerous pro-inflammatory mediators (i.e. TNF-α, CCL2, IFN-γ; **Figure 3**). Previously, descriptions of immune cell infiltration into the placental exchange region, and up-regulated placenta expression of similar pro-inflammatory mediators have been described in rodent models of LPS treatment in pregnancy (65, 66) - findings that closely mimic observations made in human placentas from cases of inflammatory PE (1, 6, 67). Even though both Poly I:C and LPS are TLR activators, Poly I:C treatment, not LPS, in pregnant mice has been shown to modulate the expression of ATP-binding cassette (ABC) transporters in the JZ leading to cell death in that region (68–70). This suggests that LPS and Poly I:C impact placenta differentially. In the TNF-α model, we did observe a reduction in labyrinth depth and fetal weight, even though we have not seen increase in CD68^+^ macrophage infiltration or in any of the tested inflammatory gene expression suggesting other mechanism or pathways may be involved. To date, very little work has been carried out to clarify region-, or function-specific deficits in placental developmental, and how they may contribute significantly to the fetal growth restriction in rodent models.

Placenta mitochondrial impairments, specifically deficits in mitochondrial respiration, are well described pathological features of PE (11–13), with our group proposing that mitochondrial dysfunction may be a common feature across all PE subclasses (13). Therefore, it is important to establish animal models that exhibit such dysfunctions. As such, any rodent PE model should likewise demonstrate dysregulated mitochondrial function. Through *ex vivo* measurements of mitochondrial oxygen consumption rates we determined that placentas from the LPS-induced PE model had reduced complex I and II driven state-III respiration rates, indicative of significant mitochondrial dysfunction considering complex I and II are the two main electron transporters in the mitochondrial ATP synthesis process (**Figures 4A, B**). TNF-α induced PE model demonstrated a moderate decrease in the mitochondrial complex-I driven state-III respiration rate (**Figure 4A**), while Poly I:C treatment did not cause any changes in mitochondrial respiration. In this study, LPS-treatment model along with a strong induction of inflammation, likewise demonstrated the most profound deficits in mitochondrial respiration. As such, it is possible that establishment of TNF-α or Poly I:C rodent models using higher doses than those tested here may in fact be capable of further compromising metabolic activity of the placenta, and therefore may more closely mimic human inflammatory PE features.

Ongoing work in our group has also identified dysregulated NAD^+^-mediated metabolic signalling and NAD^+^ depletion in placentas of human cases of inflammatory PE (14). Depletion of cellular NAD^+^ pools is associated with metabolic impairment and mitochondrial dysfunction (15, 18). NAD^+^ is essential for many redox reactions and is consumed by key metabolic signalling pathways, such as that driven by NAD^+^ dependent sirtuin deacetylases that regulate mitochondrial biogenesis and homeostasis (15, 16, 18). Inflammation-driven activation of NAD^+^ consuming enzymes and a subsequent drop in NAD^+^ levels have been described in many studies. Such dysregulated NAD^+^ metabolism not only affects mitochondrial function but also fosters inflammatory conditions, ultimately causing organ dysfunction (15, 19–22). As such, methods that help maintain NAD(H) homeostasis with NAD^+^ boosting strategies, such as treating with vitamin B3 derivatives (71), presents an attractive therapeutic strategy for chronic inflammatory diseases (15–17, 22). Our results showed that placentas from LPS treated pregnant rats exhibited a significant decrease in NAD^+^ levels, and in the NAD^+^/NADH ratio, which correlated to a reduction in mitochondrial respiration at multiple complexes, as a marker of mitochondrial function (**Figures 4D-F**). Poly I:C treatment on the other hand, did not show placenta respiratory deficiency but did demonstrate decreases in placenta NAD^+^ levels, suggesting an underlying metabolic imbalance (**Figures 4D-F**) in this model. In the TNF-α model no changes in placenta NAD(H) status was observed, further indicating this may not be the most appropriate model for the study of this distinct human PE subclass.

In conclusion, this study evaluated three inflammatory PE models, aiming to identify a model that may closely recapitulate findings described in human cases of inflammatory PE. The clinical features and impacts on fetal and placenta development differ significantly across the tested models, with the rodent model of inflammatory PE initiated by daily LPS injections from GD 13-18 deemed most suitable for this purpose. In this model, mothers demonstrated increased BP across pregnancy, evidence of proteinuria, inflammation-mediated placental dysfunction and fetal growth restriction. It is important that future mechanistic or therapeutic intervention studies for PE carried out using rodent models must thoughtfully consider the underlying etiology and pathophysiology they are aiming to study, and ensure they choose a rodent model that most closely mirrors the findings in the patient population of interest.

## Data availability statement

The original contributions presented in the study are included in the article/**Supplementary Material**, further inquiries can be directed to the corresponding authors.

## Ethics statement

The animal study was approved by University of Ottawa animal care committee (protocol# HS2923). The study was conducted in accordance with the local legislation and institutional requirements.

## Author contributions

Conceptualization, FJ, GV, KM and SB. Data collection and analysis, FJ, GV, AN-A, AG, YC. Writing, FJ. Review and editing, FJ, AG, YC, KM and SB. Project supervision and funding, KM and SB. All authors contributed to the article and approved the submitted version.

## Supplementary material

Supplementary Figure 1 Blood pressure measurement in TNF-α treated pregnant rats by telemetry. Mean blood pressure was measured from gestational day 15-19. n=4 pregnant rats/treatment group. Error bar indicates standard deviation (SD). Tumor necrosis factor (TNF-α).

Supplementary Figure 2 Representative higher resolution H&E images of placenta layers from control, TNF-α, Poly I:C and LPS groups. Degeneration of the trophoblast with LPS treatment was shown by arrows and accumulation of immune cells in the labyrinth was shown by star. Dec: decidua, Jz: junctional zone, Lab: labyrinth. Scale bar: 50µ.
