# Supplementary figures and images for: A comparison of rat models that best mimic immune-driven preeclampsia in humans
Source: Front Endocrinol (Lausanne). 2023 Sep 28;14:1219205. doi: 10.3389/fendo.2023.1219205 (PMC10569118; doi:10.3389/fendo.2023.1219205)

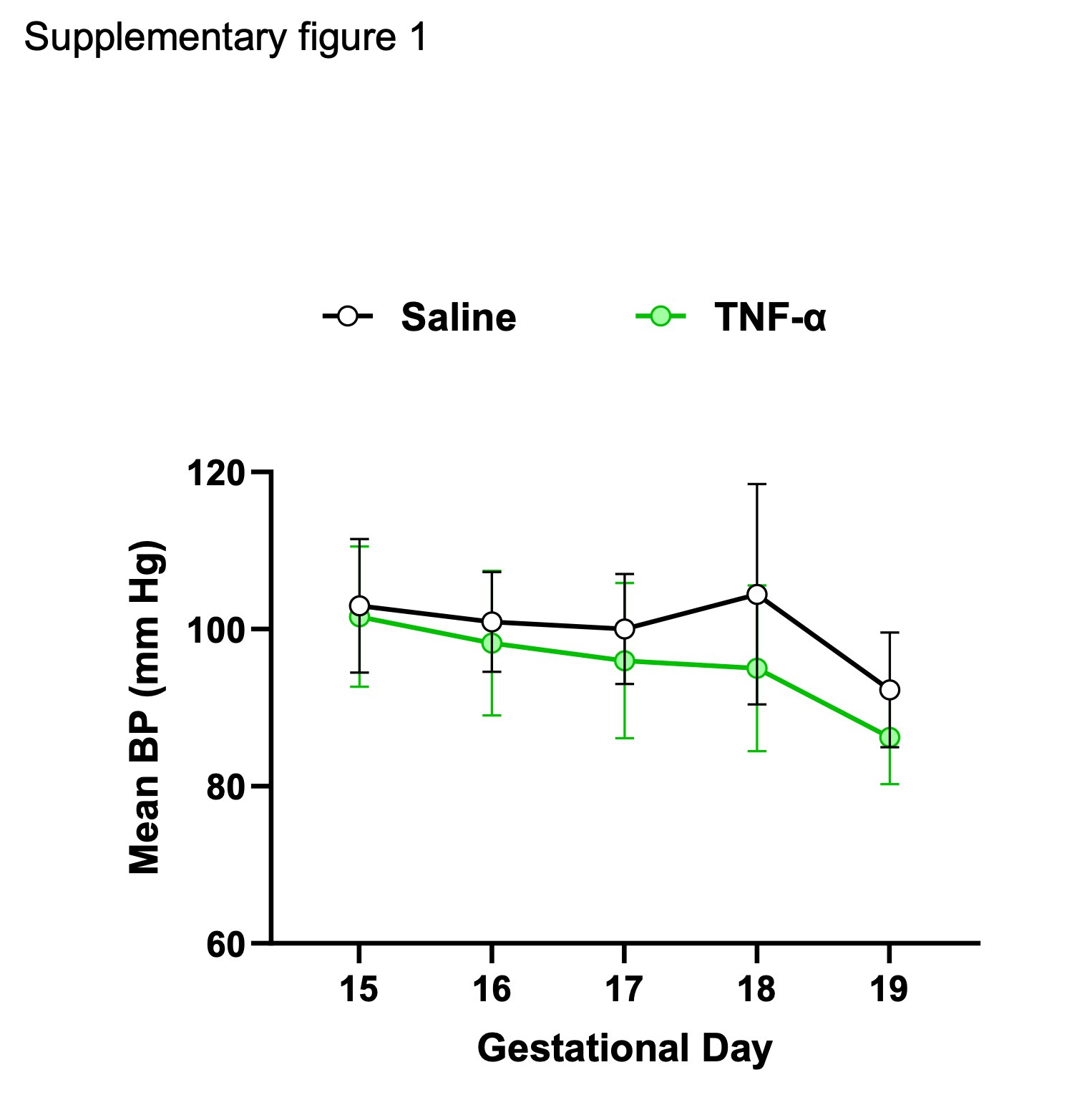

Supplement: Supplementary Figure 1 — Blood pressure measurement in TNF-α treated pregnant rats by telemetry. Mean blood pressure was measured from gestational day 15-19. n=4 pregnant rats/treatment group. Error bar indicates standard deviation (SD). Tumor necrosis factor (TNF-α). [file Image_1.jpeg]

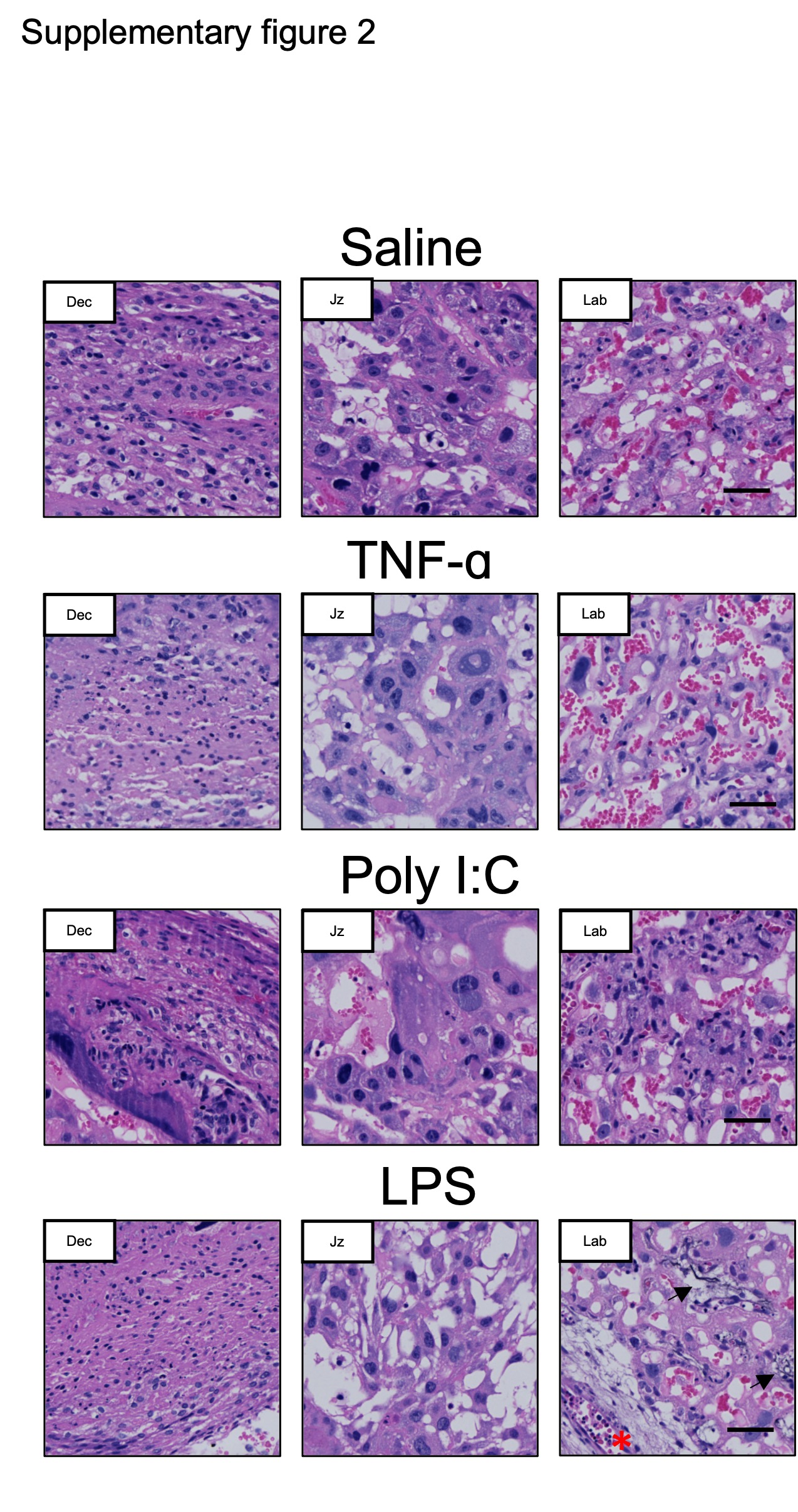

Supplement: Supplementary Figure 2 — Representative higher resolution H&E images of placenta layers from control, TNF-α, Poly I:C and LPS groups. Degeneration of the trophoblast with LPS treatment was shown by arrows and accumulation of immune cells in the labyrinth was shown by star. Dec: decidua, Jz: junctional zone, Lab: labyrinth. Scale bar: 50µ. [file Image_2.jpeg]
