# Supplementary material for: A comparison of rat models that best mimic immune-driven preeclampsia in humans
Source: Front Endocrinol (Lausanne). 2023 Sep 28;14:1219205. doi: 10.3389/fendo.2023.1219205 (PMC10569118; doi:10.3389/fendo.2023.1219205)
Supplement: Supplementary file 3 [file DataSheet_1.pdf]

Rat inflammation/pregnancy markers

TNF
NM_012675.3 https://doi.org/10.1186/s12974-015-0248-1

	Sequence (5'->3')	Template strand	Length	Start	Stop	Tm	GC%	Self complementarity	Self 3' complementarity
Forward primer	GCAGATGGGCTGTACCTTATC	Plus	21	535	555	58.23	52.38	4.00	2.00
Reverse primer	GGCTGACTTTCTCCTGGTATG	Minus	21	655	635	58.09	52.38	3.00	0.00
Product length	121bp								

IFN-g
NM_138880.3

	Sequence (5'->3')	Template strand	Length	Tm	GC%	Self complementarity	Self 3' complementarity
Forward primer	TGTTACTGCCAAGGCACACT	Plus	20	59.82	50.00	6.00	1.00
Reverse primer	ACCGTCCTTTTGCCAGTTCC	Minus	20	60.82	55.00	3.00	0.00
Product length	129bp						

MIF
NM_031051.2

	Sequence (5'->3')	Template strand	Length	Start	Stop	Tm	GC%	Self complementarity	Self 3' complementarity
Forward primer	TCACGTAGCTCAGGTCCCAG	Plus	20	4	23	60.97	60.00	4.00	2.00
Reverse primer	ACTGCGATGTACTGTGCCG	Minus	19	203	185	60.45	57.89	4.00	2.00
Product length	200bp								
Exon junction	192/193 (reverse primer) on template NM_031051.2								

PIGF
NM_053595.2

	Sequence (5'->3')	Template strand	Length	Start	Stop	Tm	GC%	Self complementarity	Self 3' complementarity

**Forward primer**	GAGGAACCCCACCTGTGATG	Plus	20	764	783	60.04	60.00	4.00	2.00
**Reverse primer**	CTTGTCCTTCCATGCCCCTT	Minus	20	956	937	59.96	55.00	4.00	0.00
**Product length**	193								
**Exon junction**	777/778 (forward primer) on template NM_053595.2								

iNOS
NM_012611.3 https://www.jimmunol.org/content/jimmunol/192/12/6009.full.pdf?with-ds=yes

	**Sequence (5'->3')**	**Template strand**	**Length**	**Start**	**Stop**	**Tm**	**GC%**	**Self complementarity**	**Self 3' complementarity**
**Forward primer**	TTCCTCAGGCTTGGGTCTTGT	Plus	21	104	124	61.33	52.38	5.00	0.00
**Reverse primer**	GGCAAGCCATGTCTGTGACTT	Minus	21	200	180	61.16	52.38	6.00	3.00
**Product length**	97								

MCP-1/CCL2
NM_031530.1 https://d-nb.info/1220602175/34

	**Sequence (5'->3')**	**Template strand**	**Length**	**Start**	**Stop**	**Tm**	**GC%**	**Self complementarity**	**Self 3' complementarity**
**Forward primer**	TGTTCACAGTTGCTGCCTGT	Plus	20	107	126	60.39	50.00	4.00	2.00
**Reverse primer**	CGACTCATTGGGATCATCT	Minus	19	215	197	54.03	47.37	5.00	3.00
**Product length**	109bp								

ICAM-1
NM_012967.1

	**Sequence (5'->3')**	**Template strand**	**Length**	**Start**	**Stop**	**Tm**	**GC%**	**Self complementarity**	**Self 3' complementarity**
**Forward primer**	CACCATCACTGTGTATTCGTTC	Plus	22	345	366	57.42	45.45	5.00	0.00
**Reverse primer**	CTACTGAGAGCTGTGTCCGC	Minus	20	475	456	60.18	60.00	4.00	2.00
**Product length**	131								
**Exon junction**	361/362 (forward primer) on template NM_012967.1								

Bactin
NM_031144.3
https://patentimages.storage.googleapis.com/9e/06/cf/6c6cde27b1c952/US20160256514A1.pdf pag.36

	Sequence (5'->3')	Template strand	Length	Start	Stop	Tm	GC%	Self complementarity	Self 3' complementarity
**Forward primer**	CTGTGTGGATTGGTGGCTCT	Plus	20	1091	1110	59.96	55.00	2.00	0.00
**Reverse primer**	GCTCAGTAACAGTCCGCCTA	Minus	20	1223	1204	59.18	55.00	3.00	2.00
**Product length**	133bp								
